# Comparison of visual estimation of ventricular systolic function with quantitative echocardiographic markers in newborn infants

**DOI:** 10.1186/s44156-026-00142-2

**Published:** 2026-10-26

**Authors:** Sujith S. Pereira, Beena Yeldo, Rachael Fleming, Saulius Satas

**Affiliations:** 1 Neonatal Unit, Homerton University Hospital, Homerton Healthcare NHS Foundation Trust, London, UK; 2 Centre for Genomics and Child Health, Barts and the London School of Medicine and Dentistry, London, UK; 3 Neonatal Unit, Aberdeen Maternity Hospital, NHS Grampian, Aberdeen, UK

**Keywords:** Ventricular systolic function, Qualitative, Quantitative, Echocardiographic markers, Point of care ultrasound, Newborn infants

## Abstract

**Background:**

Neonatologist-performed echocardiography (NPE) is increasingly practiced. NPE operators typically use visual estimation of systolic function and, if indicated, confirm function using quantitative echocardiographic markers. This exploratory study aims to compare the concordance between qualitative and quantitative echocardiographic markers among NPE and non-NPE operators.

**Methods:**

We studied a cohort of newborn infants, undergoing routine NPE by the authors at Homerton University Hospital and Aberdeen Maternity Hospital from April 2024 to October 2024. Blinded NPE and non-NPE operators independently assessed left and right ventricular systolic function visually. We compared visual estimation with standard quantitative echocardiographic markers such as shortening fraction, Simpson’s ejection fraction and global longitudinal strain for left ventricle and tricuspid annular plane systolic excursion and fractional area change for right ventricle. We examined the concordance between visual estimation and quantitative echocardiographic markers for biventricular systolic function.

**Results:**

Sixty infants with a mean (SD) gestation and birth weight of 34 (6.2) weeks and 2311 (1319) grams respectively were examined. Sensitivity testing for all echocardiographic parameters ranged from 25% to 80% whereas specificity ranged from 38% to 78%. Sensitivity increased with worsening grades of LV dysfunction defined using shortening fraction. While concordance between NPE and non-NPE operator was generally limited across most echocardiographic parameters, test-retest reliability was acceptable for several quantitative measures, including Simpson’s ejection fraction, global longitudinal strain and right ventricular fractional area change.

**Conclusion:**

Visual estimation may be a useful bedside tool for detecting severe ventricular dysfunction in newborn infants; however; quantitative echocardiographic measures are likely to improve the assessment of mild to moderate dysfunction. Further studies should confirm these findings in a larger cohort with a higher representation of infants with ventricular dysfunction.

**Supplementary Information:**

The online version contains supplementary material available at https://doi.org/10.1186/s44156-026-00142-2.

## Introduction

Neonatologist-performed echocardiography (NPE) is increasingly practiced in the neonatal unit, aiding clinicians in therapeutic intervention [1, 2]. NPE improves diagnostic precision and alters treatment course, contributing to enhanced neonatal outcomes [3–8]. The indications for NPE are varied and include but are not limited to the diagnosis of pulmonary hypertension, patent ductus arteriosus, haemodynamic evaluation, assessment of cardiac function in sepsis, and recognition of pericardial effusion and cardiac tamponade in the neonatal intensive care setting [2, 9, 10]. One of the key advantages of NPE is its ability to provide real-time information prior to initiation of therapy and monitoring response throughout treatment. This enables clinicians to monitor treatment response and effectiveness, unlike conventional parameters such as blood pressure, pulse oximetry, capillary refill time, and various blood tests which lack this ability [11–18].

Point-of-care ultrasound (POCUS) has gained considerable traction over the years in the neonatal unit [19] including assessment of cardiac filling, cardiac contractility and patent ductus arteriosus [19, 20]. It is worth noting that low-flow states, resulting from compromised cardiac contractility, can co-exist with optimal blood pressure in up to 25% of newborn infants. [21, 22] Therefore, optimal blood pressure, one of the most commonly measured cardiovascular parameters, would not guarantee adequate end organ perfusion. In sepsis, hypoxia and cardiomyopathies, assessing cardiac contractility is crucial [23–26]. Hence, it is imperative that clinicians promptly diagnose low flow states caused by poor cardiac contractility in order to swiftly administer interventions. Additionally, clinical practice is evolving from a generalised approach towards a more individualised and physiology-driven model of care, whereby POCUS is increasingly used for interrogating cardiac contractility, volume status and supporting the systemic evaluation of the critically ill neonate [19, 27].

Early diagnosis of poor cardiac contractility is best achieved through echocardiography [13, 16]. Well-established guidelines and recommendations exist for clinicians using NPE [10, 28, 29]. Nevertheless, the implementation of NPE is not without challenges. Time pressure, limited access to clinicians with expertise in functional echocardiography outside standard working hours, and inter-operator variability in skill and experience may all affect the accuracy and timeliness of diagnosis [28, 30, 31].

Quantitative echocardiographic markers, such as ejection fraction (EF), shortening fraction (SF) and tricuspid annular plane systolic excursion (TAPSE) performed as part of functional echocardiography requires expertise for accurate acquisition, and is time-consuming. POCUS can be used as a rapid tool to evaluate cardiac contractility in the neonatal unit. Clinicians can visually assess biventricular function using the apical 4-chamber or parasternal views, even with limited echocardiography experience. Unlike adults [32], there is a paucity of data comparing qualitative and quantitative markers for cardiac contractility in newborn infants using echocardiography.

We hypothesise that qualitative echocardiographic markers (visualising left and right ventricular systolic function) are likely to correlate with quantitative ventricular systolic echocardiographic markers (shortening fraction/ ejection fraction and tricuspid plane systolic excursion) when assessed by NPE and non-NPE operators [33].

Our primary objective was to examine the concordance between qualitative and quantitative echocardiographic markers to assess biventricular systolic function in newborn infants by NPE and non-NPE operators in this exploratory study.

The secondary objectives were; 1. To determine sensitivity, specificity, positive predictive and negative predictive values for qualitative echocardiographic markers and 2. To evaluate the reliability of echocardiographic measurements within observers.

## Methods and analysis

### Study design and setting

This prospective, observational, dual-centre cohort study investigated newborn infants who underwent NPE by the authors at Homerton University Hospital (HUH) and Aberdeen Maternity Hospital (AMH) over a 6-month period (1st April 2024 to 1st October 2024). HUH is a large tertiary medical neonatal unit serving the North Central and North East London Neonatal Network. [34] AMH is a large tertiary neonatal unit in North East Scotland and is one of the regional perinatal centres.

NPE operators: NPE examination were conducted by operators with extensive experience in neonatal echocardiography, including the assessment of both structural and functional echocardiography. The operators possess extensive expertise in neonatal cardiovascular assessment, including accreditation in congenital heart disease echocardiography, advanced academic training in neonatal cardiovascular pathophysiology, and active involvement in neonatal cardiovascular research. In addition, they contribute to local and national NPE education programmes and regularly participate in national and international meetings focussed on neonatal haemodynamics and cardiovascular care.

Non-NPE operators: Operators who have had minimal or no echocardiographic experience. Operators have limited echocardiography exposure by attending courses and in day to day clinical practice or exposure from case discussions, conferences and multi-disciplinary meetings. In this study, non-NPE operators did not receive any prior training or feedback during the study and only visually assessed echocardiograms acquired by NPE operators in the order described below.

### Study population

Inclusion criteria: Newborn infants, both inborn and outborn, who underwent NPE as part of routine care at the HUH and AMH over a 6-month period. NPE was performed at the discretion of the treating clinician for the assessment of murmur, in infants requiring inotropic support, and in infants with high oxygen requirement.

Exclusion criteria: Infants with congenital heart disease (except patent ductus arteriosus), missing data or major congenital abnormality.

### Data collection

We collected clinical data from electronic patient records (EPR) and the national neonatal database BadgerNet® (prospectively entered neonatal clinical database). Clinical diagnoses were gathered from EPR and BadgerNet® at the respective sites. In addition, we also collected gestational age, birth weight, gender, inotrope use, and type of respiratory support.

### Image acquisition

Echocardiographic images were acquired with 6 MHz and 12 MHz sector-array transducer for term and preterm infants respectively (GE, Vivid S60N, Buckinghamshire, UK for centre 1 and GE, Vivid S70N, Buckinghamshire, UK for Centre 2) using standard protocol [10] with frame rates typically ranging between 35 and 100 frames per second depending on the use of colour echocardiography. Annonymised apical four-chamber, apical two-chamber, parasternal long-axis, and parasternal short axis views were saved from the ultrasound scanner for the study. Infants receiving invasive mechanical ventilation were sedated, whereas images in spontaneously breathing infants were acquired during period when they were settled. Image quality was considered adequate for interpretation at both centres.

### Qualitative echocardiographic markers

Left ventricular systolic function:

Operators visually estimated left ventricular (LV) systolic function using a combination of apical 4-chamber and parasternal views. Approximation of the LV free wall and the septum, as well as the reduction in the LV cavity during systole were evaluated. The operator then classified LV contractility as either normal or impaired based on these visual observations.

Right ventricular systolic function:

Operators visually assessed right ventricular (RV) systolic function for vertical movement of the RV free wall, approximation of the RV free wall and the septum, and reduction in the RV cavity during systole. The operator then classified RV contractility as either normal or impaired based on these visual observations.

NPE operators assessed biventricular systolic function qualitatively for infants not cared for in their site. This ensured blinding to quantitative echocardiographic markers. Clinicians who are non-NPE operators at their sites were shown anonymised echocardiographic scans performed at the respective sites. Non-NPE operators were blinded to all clinical information including the indications for the study. Test-retest reliability was performed by NPE operator several months after initial assessment to minimise bias at the HUH site only.

### Quantitative echocardiographic markers

Echocardiographic markers performed in infants undergoing routine care align with established recommendations [10]. Biventricular systolic function was assessed using apical 4-chamber and parasternal views. The parasternal short axis view at the level of the mitral valve leaflet tips facilitated acquisition of the M-mode image for SF interrogation. Additionally, apical 2-chamber view was obtained to calculate the EF using Simpson’s biplane method [10].

The aforementioned markers were measured using both M-mode and 2-D images. An average of three readings were taken for measurement of SF and TAPSE to improve accuracy. The following echocardiographic measurements, validated in newborn infants, were routinely collected as part of standard care, utilising live images during scans and offline analysis following the scans:

Left ventricular end diastolic diameter (LVEDD)

Left ventricular end systolic diameter (LVESD)

Shortening fraction (SF)

Ejection fraction (EF)

Tricuspid annular plane systolic excursion (TAPSE)

Additional echocardiographic parameters, including 3-chamber right ventricular fractional area of change (RV FAC) [10] and two-dimensional speckle tracking echocardiography [35] to determine LV global longitudinal strain (LV GLS) when performed using standard techniques were also compared with visual estimation of right and left ventricular systolic function respectively. Concordance between quantitative echocardiographic markers were evaluated.

### Defining impaired ventricular function

LV systolic function:

SF below 26% [16, 36], EF less than 50% [37–39] and LV GLS values less negative than −20%(i.e.>−20%) [35] were pragmatic thresholds considered indicative of impaired LV systolic function consistent with commonly used neonatal echocardiographic criteria.

RV systolic function:

TAPSE [40] and RV FAC [41] <2 SD for age related values were considered impaired to account for size change and maturation of the right ventricle size.

### Sample size

Convenience sampling was adopted, with a pragmatic approach to sample size based on NPE operator availability to perform scans and the exclusion of pathology. Assuming that five babies would be assessed each month, this would result in 30 babies per site during the study period, yielding a total sample size of 60 infants.

### Statistical analyses

Continuous variables, including gestation, birth weight, and age at scan, were presented as mean (SD, range), while categorical variables, such as the proportion of infants with normal and reduced cardiac contractility, were expressed as frequency and percentage. Normally distributed continuous variables were compared between group using the independent samples t-test. Sensitivity, specificity, positive predictive and negative predictive values, were calculated. Non-NPE operator visual estimation of biventricular systolic function were compared to NPE operator derived quantitative echocardiographic markers at their respective site. Correlation between various echocardiographic markers were carried out using Pearson’s correlation.

To examine test-retest reliability within observers, intraclass correlation (ICC) was determined using 2-way mixed ANOVA, where observers were fixed factors and measurements, random factors. [42–44] Concordance between a pair of raters and the gold-standard classification was assessed using Cohen’s Kappa coefficient. [43] Disease prevalence was also examined. All statistical analyses were conducted using IBM SPSS v29 (Chicago, IL, USA), GraphPad Prism version 10.2.0 and Microsoft excel.

Patient and public were not involved in this study design. This study received approval from the Health and Care Research Wales (HCRW) Research Ethics Committee and Health Research Authority (HRA) (reference 23/HRA/3066). We also sought Caldicott Guardian approval (Registration number Z8547986) for the study site at AMH, NHS Grampian. The local Research and Innovation (R&I) department of Homerton Healthcare NHS Foundation Trust and AMH, NHS Grampian also reviewed and approved this study.

## Results

Sixty infants with a mean gestation and birth weight of 34 (6.2) weeks and 2311 (1319) grams respectively were studied. Infants in centre 1 had significantly lower gestational age and birth weights than infants in centre 2 (Table 1). Invasively ventilated infants were substantially higher in centre 1 than 2 (80.0% versus 46.7%) as was inotrope use; however this difference was not statistically significant.

**Table 1:** Clinical characteristics of the infants

Clinical parameters		Centre 1 (HUH) N = 30	Centre 2 (AMH) N = 30	*p***-value**
Gestation, weeks		31.7 (6.0)	36.2 (5.6)	0.005
Birth weight, grams		1874.6 (1227.1)	2748.5 (1281.1)	0.009
Gestation at scan, weeks		34.1 (6.1)	37.4 (4.9)	0.025
Weight at scan, grams		2124.2 (1288.1)	2811.5 (1202.7)	0.037
Age at scan, days (SD, range)		16.9 (30.9,0 − 170.5)	8.8 (13.9,0–61.3)	0.201
Respiratory support, n(%)	MV	18 (60%)	11 (36.7%)	0.008
	HFOV	6 (20%)	3 (10%)	
	NIV	2 (6.7%)	-	
	BU	4 (13.3%)	16 (53.3%)	
Mean airway pressure, cms		9.7 (3.9)	8.3 (4.7)	0.333
FiO2		0.4 (0.2)	0.4 (0.3)	0.634
Inotropes, n(%)		5 (16.7%)	2 (7%)	0.106

MV – conventional mechanical ventilation, HFOV – high frequency oscillatory ventilation, NIV – non-invasive ventilation, BU – breathing unaided, FiO2 – fraction of inspired oxygen. Unless specified, all figures represent mean (SD)

Concordance between NPE and non-NPE operators varied across the echocardiographic parameters examined (Table 2, supplementary table 1). The prevalence of ventricular dysfunction in the study cohort was low, particularly for left ventricular parameters (supplementary table 2).

**Table 2:** Concordance between qualitative and quantitative echocardiographic markers for the different NPE and non-NPE raters using Cohen’s Kappa

Visual estimation	Echocardiographic markers	Rater 1 (NPE)	Rater 2 (NPE)	Rater 3 (Non-NPE)	Rater 4 (Non-NPE)
LV	Shortening fraction	67.0%* 0.21 (0.14–0.28)	54.0%* 0.08 (−0.02–0.17)	69.0%* 0.05 (−0.01–0.10)	53.0%* 0.01 (−0.07–0.10)
	Simpson’s biplane EF	30.0%* −0.03 (−0.16–0.10)	57.0%* 0.17 (0.07–0.28)	48.0%* −0.14 (−0.23 – −0.05)	43.0%* 0.03 (−0.09–0.14)
	Global longitudinal strain	48.0%* 0.00 (−0.11–0.11)	50.0%* 0.11 (−0.02–0.24)	61.0%* −0.03 (−0.12–0.05)	52.0% 0.05 (−0.05–0.16)
RV	TAPSE	52.0%* 0.09 (−0.01–0.19)	57.0%* 0.01 (−0.08–0.10)	65.0%* 0.29 (0.19–0.38)	56.0%* 0.10 (0.01–0.20)
	FAC	-	43.0%* −0.14 (−0.27 - −0.01)	79.0%* 0.55 (0.45–0.66)	-

*Represents raw percent agreement, Values represent Kappa (95% confidence intervals). Shaded and unshaded columns show comparison between stated raters (NPE rater 2 versus non-NPE rater 3 compared and NPE rater 1 versus non-NPE rater 4 compared). LV – left ventricle, RV – right ventricle, EF – ejection fraction, TAPSE – tricuspid annular plane systolic excursion, FAC – fractional area change

**Fig. 1:**
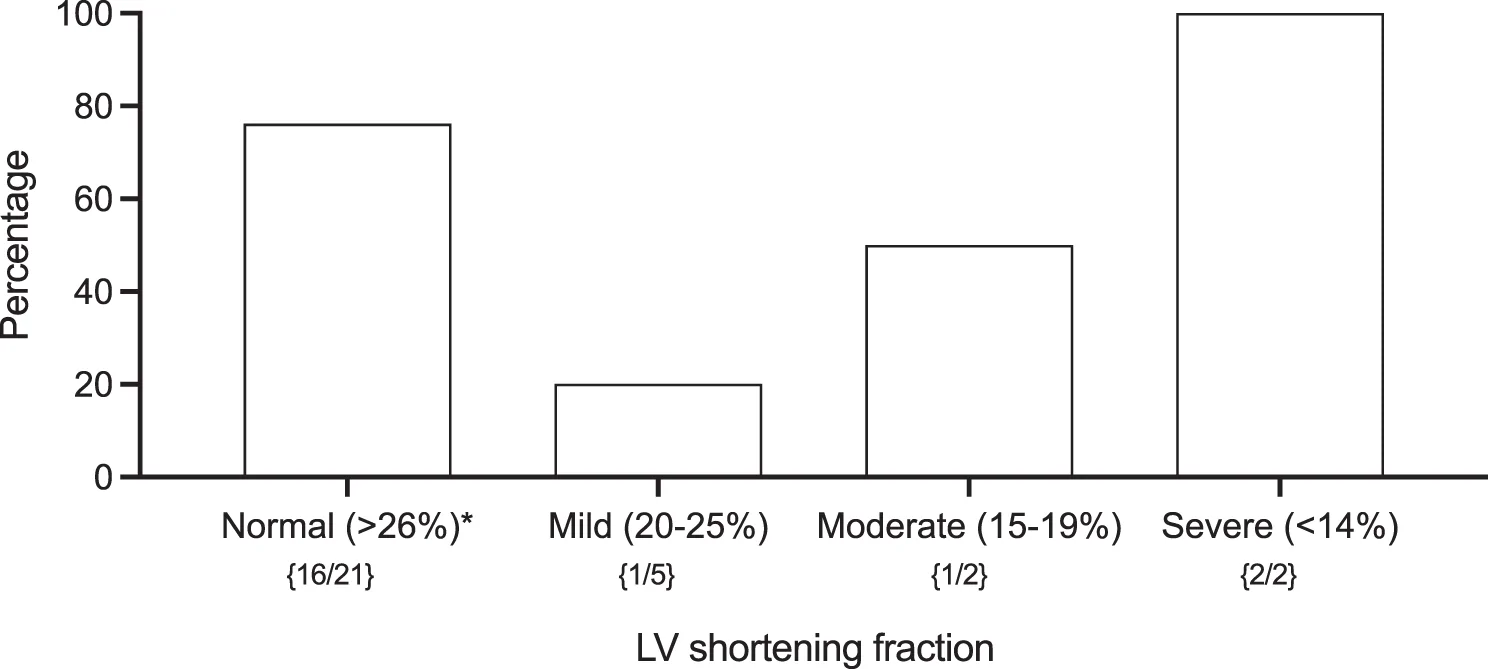
Specificity* and sensitivity (for rater 1) for the identification of normal and impaired LV function across worsening grades of LV dysfunction, as defined by SF. [16, 45] figures in parentheses represent {n/N}. LV – left ventricle, SF – shortening fraction

**Fig. 2:**
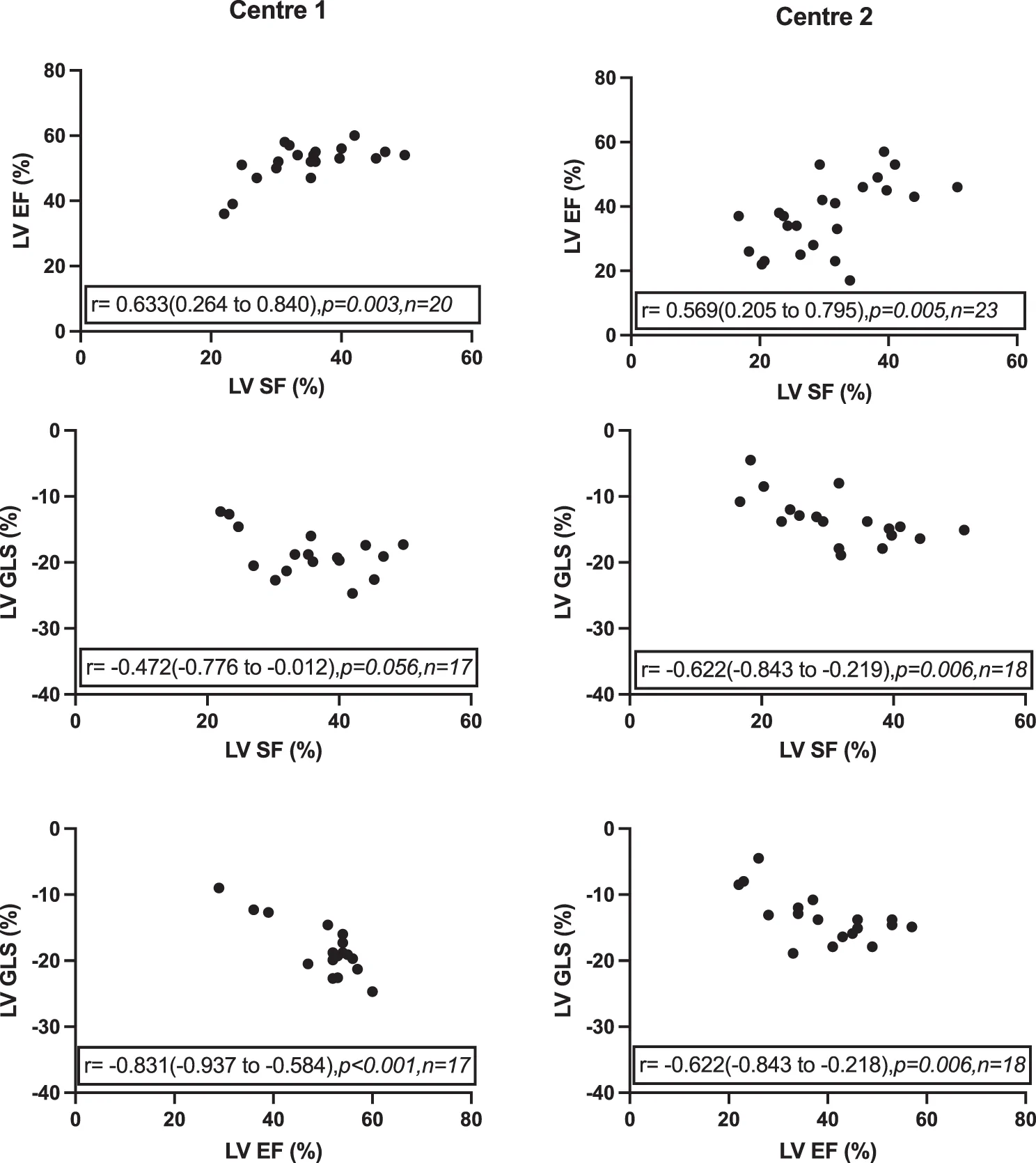
Correlation (with 95% confidence intervals) between various quantitative echocardiographic markers

Sensitivity testing for LV parameters ranged from 25% to 44%, for rater 1 (NPE operator) and rater 3 (non-NPE operator), whereas specificity ranged from 56% to 76% (Table 3). Overall positive and negative likelihood ratios did not demonstrate a strong association with the presence or absence of disease (supplementary table 3). Specificity was higher for normal LV function and sensitivity increased with worsening grades of LV dysfunction (from 20% for mild dysfunction to 100% for severe dysfunction) defined using SF for rater 1 (Fig. 1). Correlation between RV FAC and TAPSE showed a positive correlation which was statistically insignificant (*r* = 0.51, *p* = 0.07). There was a significant correlation for the majority of quantitative echocardiographic parameters studied (Fig. 2). Concordance between quantitative echocardiographic markers among NPE raters are shown in Table 4.

**Table 3:** Various diagnostic tests compared between the raters for all infants in the study

Visual estimation	Echocardiographic markers/ Raters	Sensitivity				Specificity			
		1	2	3	4	1	2	3	4
LV	SF	44 (12–77)	67 (13–100)	33 (0–87)	44 (12–77)	76 (58–94)	52 (32–73)	74 (56–92)	57 (36–78)
	EF	25 (6–44)	71 (38–100)	29 (0–62)	40 (19–61)	67 (13–100)	50 (26–75)	56 (32–81)	67 (13–100)
	GLS	33 (7–60)	75 (33–100)	25 (0–67)	50 (22–78)	67 (36–97)	43 (17–69)	71 (48–95)	56 (23–88)
RV	TAPSE	73 (56–90)	40 (20–60)	80 (64–96)	55 (36–73)	38 (9–66)	61 (18–100)	61 (18–100)	56 (27–86)
	FAC	-	40 (0–83)	80 (45–100)	-	-	44 (12–77)	78 (51–100)	-

LV – left ventricle, RV – right ventricle, SF – shortening fraction, EF – ejection fraction, GLS – global longitudinal strain, TAPSE – tricuspid annular plane systolic excursion, FAC – fractional area change. All figures represent % (95% confidence intervals)

**Table 4:** Concordance between the various echocardiographic markers among the two NPE raters

Ventricle	Echocardiographic markers	Rater 1	Rater 2
Left ventricle	LV SF and EF	0.85* 0.48 (0.44–0.53)	0.43* 0.12 (−0.01–0.25)
	LV SF and LV GLS	1.00* 1 (1)	0.62* 0.28 (0.17–0.39)
	LV EF and LV GLS	0.85* 0.48 (0.44–0.53)	0.43* 0.12 (−0.01–0.25)
Right ventricle	TAPSE and RV FAC	0.91* 0.81 (0.73–0.90)	-

*Represents raw percent agreement. Values represent Kappa (95% confidence interval). LV – left ventricle, RV – right ventricle, SF – shortening fraction, EF – ejection fraction, GLS – global longitudinal strain, TAPSE – tricuspid annular plane systolic excursion, FAC – fractional area change

### Test re-test reliability

Test re-test reliability for the various echocardiographic parameters for rater 1 are shown in Table 5. Rater 1 concordance between qualitative echocardiographic markers for left ventricle revealed a percent agreement of 0.77 and Kappa coefficient of 0.44 (95% confidence interval 0.38–0.51) whereas for the right ventricle, percent agreement was 0.97 and Kappa coefficient was 0.93 (0.90–0.96).

**Table 5:** Test re-test reliability for rater 1. LV – left ventricle, RV – right ventricle, EF – ejection fraction, TAPSE – tricuspid annular plane systolic excursion, FAC – fractional area change

Visual estimation	Echocardiographic markers	Intraclass correlation (95% CI)	*p***-value**
LV	Shortening fraction	0.45 (−0.16–0.75)	0.060
	Simpson’s biplane EF	0.61 (0.09–0.84)	0.016
	Global longitudinal strain	0.64 (0.05–0.86)	0.021
RV	TAPSE	0.52 (−0.05–0.79)	0.033
	FAC	0.83 (0.44–0.95)	0.002

## Discussion

In this prospective, dual-centre exploratory study, we evaluated the concordance between NPE and non-NPE operators in the qualitative and quantitative assessment of the LV and RV function in newborn infants. Our findings demonstrate that visual estimation may be a useful bedside tool for identifying severe ventricular dysfunction, supporting its role in the rapid clinical assessment of critically ill neonates. However, concordance was lower for the detection of mild to moderate dysfunction and for several quantitative measures, highlighting the challenges associated with subjective assessment and underscoring the need for standardised training, objective functional parameters, and continued efforts to improve reproducibility in neonatal echocardiography.

Sensitivity for all the LV parameters varied across operators in this study. We observed that sensitivity for LV dysfunction, as measured using SF, increased with worsening dysfunction. Clinicians were more likely to accurately identify ventricular systolic function at the extremes, corresponding to a very normal or severely impaired function. Our finding of low concordance between visual estimation of LV systolic function and quantitative echocardiographic markers has been reported by other investigators examining different patient populations. Martin-Flores *et al.,* using animal studies reported the success rate for identifying moderate SF reduction (SF 15–19%) was 50% to 54% whereas severe reduction (SF < 15%) was correctly identified 92% to 96% of the time among practitioners of varying experience [46], a result consistent with our findings. Additionally, Cole and colleagues in adult patients, demonstrated that agreement between operators when visually grading LV function into categories, was 50%. [47] Lang et al. urged caution in visual estimation of LV function [48] and in their 2015 update, recommended that LV systolic function should be routinely assessed using EF. [49] Sievers et al. compared visual estimation of LV function with EF measured using magnetic resonance imaging and found that visual estimation significantly underestimated LV EF, particularly in patients with normal function. They concluded that visual estimation may be used for rapid assessment in clinical practice when high accuracy is of less concern and encouraged quantitative assessment for accurate analysis. [50] Finally, McNamara et al. in their recently published guidelines, suggested that qualitative evaluation of cardiac function should be limited to critical emergencies given its limited ability to detect mild or moderate dysfunction.[10]

Specificity was comparable between NPE and non-NPE operators for the assessment of left ventricular systolic function, suggesting that visually assessed normal cardiac function can be reliably identified in hearts with preserved performance. These findings are consistent with more recent work by Zayani and colleagues, who showed that visual estimation of EF compared favourably with echocardiography-derived EF in a cohort of children where nearly 90% had normal ventricular function.[51]

Sensitivity and specificity for RV parameters were relatively higher for both NPE and non-NPE operators in this study. Smith and colleagues who examined accuracy and reliability of qualitative echocardiographic markers for RV size and function among experts and novice practitioners, found significant variability in qualitative assessment. [52] Ling et al. assessed concordance between qualitative and quantitative RV echocardiographic and cardiac magnetic resonance (CMR) imaging markers in adults. They found quantitative measurements improved both accuracy and inter-observer agreement. [53] In their work, Narayan and colleagues examined a small cohort of 8 patients and demonstrated no concordance between qualitative markers and quantitative RV markers examined using CMR. [54] They concluded that quantitative echocardiographic assessments using RV FAC and TAPSE offers potentially more accurate measurements similar to those derived using CMR. Furthermore, visual estimation for RV function may have additional challenges owing to its complex structural geometry, muscle fibre orientation and response to afterload. These can predispose to inaccuracies, making quantitative assessments the preferred method to accurately interrogate RV function.[55]

The low concordance between qualitative and quantitative assessment observed in this study could be attributed to several factors. The human eye can reliably identify very normal or very abnormal heart function, but infants with borderline cardiac dysfunction risk misclassification, an issue which could be alleviated with the emergence of artificial intelligence. [56, 57] We did not use ventricular contrast, shown to improve identification of ventricular assessment [58], as we wanted to simulate real world practice in the neonatal unit. We did not prescribe a time limit on visual assessment per image to mirror reality. It is possible that spending more time assessing an image may improve assessment accuracy because of a learning effect although it remains unclear which images may benefit from this. Atrio-ventricular valvar regurgitation can give a false impression of optimal ventricular systolic function. Finally, suboptimal images, operator experience and artefacts are other factors which could contribute to poor concordance.

This hypothesis generating study has several limitations that should be considered when interpreting the findings. The sample size was relatively small, reflecting the exploratory nature of this study, and the prevalence of significant cardiac dysfunction was low. However, the inclusion of a largely unselected neonatal population provides insight into the performance of echocardiographic assessment in a real-world clinical setting. Furthermore, the use of convenience sampling allowed evaluation across the spectrum of cases encountered in routine practice, although it limited control over the prevalence and severity of pathology assessed by the NPE operators. The cohorts studied were heterogenous. Infants in one centre were significantly more premature, needing more respiratory support and required minimal inotropic support. The modest use of inotropes limited our ability to investigate its effect on visual estimation. In preterm infants, surfactant deficient lung disease of varying severity may necessitate respiratory support, including invasive ventilation, which can alter preload and subsequently impact the echocardiographic measures studied. While this study allowed us to evaluate some infants with severe dysfunction, our ability to examine more infants needing invasive respiratory support with mild to moderate cardiac dysfunction was restricted. In order to ensure blinding and reduce bias, we were unable to compare NPE versus NPE and non-NPE versus non-NPE operators. Although we used quantitative echocardiographic markers for this study, these are not without limitations. These markers can be imperfect especially when exposed to the dynamic physiology from intracardiac shunts and altered loading conditions seen in transitional circulation, a unique characteristic of the studied neonatal population, adding an additional layer of complexity. [59] This study did not account for the haemodynamic significance of patent ductus arteriosus, which may alter loading conditions and potentially influence quantitative echocardiographic measurements. Invasively ventilated infants were more likely to receive sedation and therefore likely to have superior image quality. Active infants can affect diagnostic precision [60] and influence visual grading of EF [47] whereas sedation may affect image quality. [61] NPE operators acquired the images whereas non-NPE operators interpreted these images. The acquisition skill itself can have an impact on echocardiographic assessment. In this exploratory study, LV systolic function was classified as a binary outcome (normal or impaired) to evaluate the operators’ ability to identify ventricular dysfunction. Further stratification according to severity may have provided additional information and potentially influenced concordance. This could be explored in larger studies enriched for infants with ventricular dysfunction, which would allow meaningful differences between severity grades to be assessed. Among the echocardiographic parameters used, data completeness was higher for SF and TAPSE for LV and RV parameters respectively when compared to other echocardiographic markers. Test-retest analysis was available for only one NPE operator, limiting the assessment of the reproducibility of the echocardiographic markers evaluated. Lastly, NPE operators may have had greater experience in both image acquisition and interpretation, which may have influenced image quality, accuracy and reproducibility of qualitative and quantitative assessments. It is therefore difficult to disentangle the effects of acquisition expertise from interpretative experience. Differences in clinical experience, prior echocardiographic exposure and NPE training may also have contributed to differences between NPE and non-NPE operators.

Our study strengths include comparison of LV and RV functional assessment using qualitative evaluation across multiple echocardiographic views and commonly used quantitative echocardiographic. We averaged three measurements for SF and TAPSE, thereby improving accuracy. NPE operators visually assessed each other’s echocardiographic images, thus ensuring blinding. NPE operator on one site re-evaluated the anonymised echocardiograms 8 months later, which minimised bias. We studied a recent cohort of infants, reflecting real-world practice in the neonatal unit. Lastly, this is a clinically relevant study and timely as neonatal haemodynamic assessments and cardiac POCUS continue to expand across neonatal units.

In contemporary neonatal practice, visual estimation of cardiac function remains a pragmatic bedside tool, particularly for the rapid identification of severe dysfunction where concordance with quantitative measures appears greatest. A representative example is the assessment of cardiac function in a neonate with sepsis, where rapid evaluation may facilitate timely optimisation of therapy. Another potential application is in hypoxic ischaemic encephalopathy with pulmonary hypertension, where visual estimation may aid in ruling out severe LV dysfunction, a key contraindication to pulmonary vasodilator therapy. Our findings suggest that visual estimation alone is likely insufficient for detecting mild or borderline impairment, and reliance on qualitative evaluation alone in such cases risks under-recognition of clinically relevant dysfunction. Where feasible, quantitative echocardiographic measures should therefore be incorporated into assessment, while recognising their own limitations in the context of neonatal physiology and loading conditions. Importantly, in real world settings where access to comprehensive quantification may be constrained, visual assessment should be considered a screening rather than a definitive tool, prompting further evaluation with persisting clinical concerns. The findings of this work also highlight the need for training programmes to emphasise not only acquisition of echocardiography skills but also an understanding of the limitations of visual estimation, supporting a more integrated approach that combines clinical, serial assessment and quantitative assessment to guide decision making.

In conclusion, visual estimation may have potential as an adjunctive bedside assessment for recognising ventricular dysfunction in newborn infants, particularly in critically ill infants where rapid clinical assessment is required. However, its reliability is reduced in the assessment of mild to moderate dysfunction and in detecting subtle changes in ventricular performance over time. These findings support the incorporation of quantitative echocardiographic measurements alongside qualitative assessment to improve diagnostic accuracy, particularly when clinical concerns persist, or serial evaluation is required. Future studies should explore this confirmation in a larger cohort enriched for ventricular dysfunction, compare the effects of inotropes on regional myocardial function and their potential influence on qualitative echocardiographic assessment, with the aim of further refining the evaluation of ventricular function in newborn infants.

## Electronic supplementary material

Below is the link to the electronic supplementary material.

Supplementary Material 1

## Data Availability

All data supporting the findings of this study are available within the paper.
