# Supplementary material for: Comparison of visual estimation of ventricular systolic function with quantitative echocardiographic markers in newborn infants
Source: Echo Res Pract. 2026 Oct 26;13:40. doi: 10.1186/s44156-026-00142-2 (PMC13700331; doi:10.1186/s44156-026-00142-2)
Supplement: Supplementary file 1 [file 44156_2026_142_MOESM1_ESM.docx]

**Supplementary data**

Supplementary table 1. Number of studies that were examined for each echocardiographic parameter among the various raters.

| **Visual estimation** | **Echocardiographic markers** | **Rater 1**  **(NPE)** | **Rater 2**  **(NPE)** | **Rater 3**  **(Non-NPE)** | **Rater 4**  **(Non-NPE)** |
| --- | --- | --- | --- | --- | --- |
|  |  | Echocardiographic studies (n) | | | |
| LV | Shortening fraction | 30 | 26 | 26 | 30 |
|  | Simpson’s biplane EF | 23 | 23 | 23 | 23 |
|  | Global longitudinal strain | 21 | 18 | 18 | 21 |
| RV | TAPSE | 27 | 23 | 23 | 27 |
|  | FAC | - | 14 | 14 | - |

Supplementary table 2. Disease prevalence among the various echocardiographic quantitative markers.

| **Visual estimation** | **Echocardiographic markers** | **Rater 1**  **(NPE)** | **Rater 2**  **(NPE)** | **Rater 3**  **(Non-NPE)** | **Rater 4**  **(Non-NPE)** |
| --- | --- | --- | --- | --- | --- |
|  |  | Disease prevalence (%) | | | |
| LV | Shortening fraction | 23.8% | 47.8% | 26.1% | 42.9% |
|  | Simpson’s biplane EF | 33.3% | 50.0% | 43.8% | 33.3% |
|  | Global longitudinal strain | 33.3% | 57.1% | 28.6% | 44.4% |
| RV | TAPSE | 72.7% | 40.0% | 80.0% | 54.5% |
|  | FAC | - | 55.6% | 22.2% | - |

Supplementary table 3. Various diagnostic tests compared between the raters for all infants in the study. LV – left ventricle, RV – right ventricle, SF – shortening fraction, EF – ejection fraction, GLS – global longitudinal strain, TAPSE – tricuspid annular plane systolic excursion, FAC – fractional area change, PPV – positive predictive value, NPV – negative predictive value, LR+ - positive likelihood ratio, LR- - negative likelihood ratio. All figures represent % except values in LR+ and LR-.

| **Visual estimation** | **Echocardiographic markers/ Raters** | **PPV** | | | | **NPV** | | | | **LR+** | | | | **LR-** | | | |
| --- | --- | --- | --- | --- | --- | --- | --- | --- | --- | --- | --- | --- | --- | --- | --- | --- | --- |
|  |  | **1** | **2** | **3** | **4** | **1** | **2** | **3** | **4** | **1** | **2** | **3** | **4** | **1** | **2** | **3** | **4** |
| LV | SF | 44.4 | 15.4 | 14.3 | 30.8 | 76.2 | 92.3 | 89.5 | 70.6 | 1.9 | 1.4 | 1.3 | 1.1 | 0.7 | 0.6 | 0.9 | 0.9 |
|  | EF | 83.3 | 38.5 | 22.2 | 88.9 | 11.8 | 80.0 | 64.3 | 14.3 | 0.8 | 1.4 | 0.7 | 1.2 | 1.1 | 0.6 | 1.3 | 0.9 |
|  | GLS | 57.1 | 27.3 | 20.2 | 60.0 | 42.9 | 85.7 | 76.9 | 45.5 | 1.0 | 1.3 | 0.9 | 1.1 | 1.0 | 0.6 | 1.1 | 0.9 |
| RV | TAPSE | 44.4 | 22.2 | 36.4 | 46.2 | 66.7 | 78.6 | 91.7 | 64.3 | 1.2 | 1.0 | 2.1 | 1.2 | 0.7 | 1.0 | 0.3 | 0.8 |
|  | FAC | - | 28.6 | 66.7 | - | - | 57.1 | 87.5 | - | - | 0.7 | 3.6 | - | - | 1.4 | 0.3 | - |
